# Prediction, Synthesis and Evaluation of a Synthetic Peptide as an Enzyme-Linked Immunosorbent Assay (ELISA) Candidate for Screening of Bovine Antibodies against *Theileria annulata*

**DOI:** 10.3390/microorganisms11112663

**Published:** 2023-10-30

**Authors:** Prasanta Kumar Koustasa Mishra, Anupama Jena, Souti Prasad Sarkhel, Sujit Kumar Behera, Annada Das, Thankappan Sabarinath, Dayanidhi Jena, Kruti Debnath Mandal, Adhikari Sahu, Anshuman Kumar, Vinod Kumar, Rahul Ganpatrao Kadam, Srinivas Sathapathy, Thavitiki Prasada Rao

**Affiliations:** 1 Unit of Teaching Veterinary Clinical Complex, Faculty of Veterinary and Animal Sciences, Institute of Agricultural Sciences, RGSC, Banaras Hindu University, Mirzapur 231001, Uttar Pradesh, India; dayanidhi5@bhu.ac.in (D.J.); krutimandal@bhu.ac.in (K.D.M.); vet.vk134@gmail.com (V.K.); rahulkadam@bhu.ac.in (R.G.K.); 2 Fisheries and Animal Resource Development Department, Bhubaneswar 751001, Odisha, India; anupamajenap62@gmail.com (A.J.); dasannada.555@gmail.com (A.D.); 3 Department of Veterinary Parasitology, Faculty of Veterinary and Animal Sciences, Institute of Agricultural Sciences, RGSC, Banaras Hindu University, Mirzapur 231001, Uttar Pradesh, India; souti.sarkhel@bhu.ac.in; 4 Department of Epidemiology & Public Health, Central University of Tamil Nadu, Tiruvarur 610001, Tamil Nadu, India; sujitbehra@gmail.com; 5 Clinical Bacteriological Laboratory, Indian Council of Agricultural Research—Indian Veterinary Research Institute, Nainital 263138, Uttarakhand, India; drsabari143ivri@gmail.com; 6 Department of Veterinary Parasitology, College of Veterinary Science and Animal Husbandry, Odisha University of Agriculture and Technology, Bhubaneswar 751003, Odisha, India; asahu@ouat.ac.in; 7 Department of Animal Genetics and Breeding, Faculty of Veterinary and Animal Sciences, Institute of Agricultural Sciences, RGSC, Banaras Hindu University, Mirzapur 231001, Uttar Pradesh, India; anshuman@bhu.ac.in; 8 Department of Veterinary Anatomy and Histology, College of Veterinary Science and Animal Husbandry, Odisha University of Agriculture and Technology, Bhubaneswar 751003, Odisha, India; srinivassathapathy@ouat.ac.in; 9 Department of Veterinary Physiology and Biochemistry, Sri Venkateswara Veterinary University, College of Veterinary Science, Garividi 535101, Andhra Pradesh, India; prasadmvsc@gmail.com

**Keywords:** tick-borne diseases, *Theileria annulata*, ELISA, peptide antigen, TaSP

## Abstract

Tick-borne diseases (TBDs) of livestock are endemic across various parts of tropical countries. Theileriosis is one such economically important TBD, caused by the Theileriidae family of organisms, which is transmitted by ticks. *Theileria annulata*, the causative agent of tropical theileriosis, contributes a significant loss to the dairy sector by causing anorexia, high fever, anemia, inflammatory changes in vital organs and icterus, thus, a loss in milk yield. Though vaccines are available, their protective efficacy is not absolute, and treatment is limited to early diagnosis of the causative agent. Routinely, microscopic identification of piroplasms in the erythrocytes (Giemsa-stained) of infected animals or schizonts in lymph node biopsies are practiced for diagnosis. PCR-based techniques (multiplex, uniplex, nested and real-time) have been reported to perform well in diagnosing active infection. Several attempts have been made using serological assays like Dot blot, ELISA and ICT, but the results were of variable sensitivity and specificity. Recombinant proteins like the *Theileria annulata* merozoite surface antigen (Tams1) and *Theileria annulata* surface protein (TaSP) have been explored as antigenic candidates for these assays. In the present study, we predicted an immunogenic peptide, i.e., TaSP-34, from the TaSP using various computational tools. The predicted peptide was custom synthesized. The diagnostic potential of the peptide was assessed by indirect plate ELISA to detect the bovine-IgM against *Theileria annulata*. Alongside, a recombinant truncated TaSP (rTaSP(tr)) was expressed and purified, which was used to compare the performance of the peptide as a diagnostic candidate. The IgM-based peptide ELISA was 100% sensitive and 92.77% specific as compared to PCR (Tams1 targeting), while 98.04% sensitivity and 97.44% specificity were observed in comparison with rTaSP(tr) ELISA. Almost perfect agreement between peptide ELISA and Tams1 PCR was observed with a Cohen’s kappa coefficient (κ-value) of 0.901 and agreement of 95.31%. Further, the κ-value between the peptide ELISA and rTaSP(tr) ELISA was found to be 0.95, and the agreement was 97.65%, which shows a good correlation between the two tests. The findings suggest that the TaSP-34 peptide can be an efficient and new-generation diagnostic candidate for the diagnosis of *T. annulata.* Furthermore, the peptide can be synthesized commercially at a larger scale and can be a cost-effective alternative for the protein-based diagnostic candidates for *T. annulata.*

## 1. Introduction

Bovine tropical theileriosis caused by *Theileria annulata* is one of the major tick-borne hemoprotozoan parasitoses in the tropics and subtropics [1]. Regions of southern Europe, Asia and northern Africa experience the endemicity of the disease in local Zebu cattle breeds. Moreover, cross-bred cattle are highly susceptible to *T. annulata* infection across the prevalent zones [2]. Clinical theileriosis cases are mostly observed in exotic and cross-bred bovines, whereas incidents of immunosuppression or stress may precipitate the disease in the indigenous carrier cattle stocks [3]. The widespread abundance of the hard tick *Hyalomma anatolicum anatolicum* vector is often attributed as the primary factor for the high endemic stability of the disease, which contributes to significant economic damages of around USD 498.7 annually in India [4]. In theileriosis endemic pockets, asymptomatic carrier cattle play a major role in the transmission of the pathogen to both locally abundant tick vectors and susceptible animal populations. Routine screening of host animals still holds good value for determining the status of seroprevalence and the endemic stability of the disease in order to devise a cost-effective control program [5]. The serology-based pen-side tests offer unparalleled advantages over other lab-based approaches due to their increased mobility, rapidity, high population coverage and involvement of low-cost inputs [6]. The available serological diagnostic tools use various recombinant proteins—i.e., Tams1, Spm2 and TaSP—as the antigenic candidates [7,8,9,10,11,12]; however, cross-reactivity with the antibodies of other hemoprotozoan diseases limits their wider application [13]. This warrants the identification of novel antigenic candidates, which can have higher detection efficiency with more specificity. An approach was made to predict new proteins of *Theileria annulata* with immunogenic potential. Four new protein candidates were also expressed and evaluated but found to be inferior to the TaSP in terms of specificity and sensitivity [14]. Newer classes of detection moieties like peptides, aptamers and peptide nucleic acids may address the shortcomings and may replace the traditional protein candidates. Peptide-based diagnostic tools offer several advantages over protein-based candidates in terms of shelf life, production cost, storage condition, sensitivity and specificity [15]. In the present study, we tried to predict a potential epitope stretch of TaSP and evaluated its potential as an ELISA candidate.

## 2. Materials and Methods

### 2.1. Blood Samples, Microscopy and Isolation of Total Blood DNA

Bovine blood samples were collected from the repository of the diagnostic section of the Unit of Veterinary Clinical Complex (VCC), Faculty of Veterinary and Animal Sciences, Banaras Hindu University. Necessary consent was obtained from the animals’ owners and clinician. The bovine blood samples were not purposefully collected; rather, leftover routine samples submitted to the diagnostic laboratory of the VCC for diagnosis (as requested by clinicians) were used in the study. A total of 128 samples were obtained from the repository. Blood samples collected in vacutainer tubes (with EDTA, 1.8 mg/mL) were harvested for microscopy and isolation of plasma and whole-blood DNA. A maximum gap of 2 days was left between the dates of blood collection and processing, and during this time, the blood was stored at 4 °C. Giemsa-stained blood smears were prepared and piroplasms were searched under bright field microscopy at 100× magnification. A total of 50 different microscopic fields were searched by a single observer. A total of 1 mL of blood was harvested for plasma. From each sample, 300–400 µL of plasma was collected. The plasma samples were labeled and stored at −20 °C until further use. Two hundred microliters of unclotted blood was used for isolation of total genomic DNA. QIAamp DNA Blood mini kit (Karlsruhe, Germany) was used for isolation of the DNA. Manufacturer’s instructions were invariably followed during the isolation. The quality of the isolated DNA was checked by NanoDrop™ 2000.

### 2.2. Screening of the Samples by Polymerase Chain Reaction

Primers targeting *Tams1* gene of *T. annulata* were custom synthesized as per the sequence given by Ganguly et al. 2019 [16]. The sequence of the primers used for amplification is as follows: forward primer Tams1 (5′-3′)—TGAGTTAACTGTCGCGGATG; reverse primer Tams1 (5′-3′)—TGGGCAGGGTGAAGATTAAG. GoTaq^®^ G2 Green Master Mix (Promega, Madison, WI, USA) was used for preparing the reaction mixture. A Biorad T-100 thermocycler was used for PCR amplification. The amplification was performed until reaching 32 cycles with a reaction volume of 25 µL. The amplicons were analyzed on a 2.5% agarose gel with a 50 bp ladder (PG210-500DI, Nex-Gen). A total of 1 µL of the whole blood DNA, isolated from each of the different samples, was used as template. The gel was visualized by GELSTAN4x transilluminator (Mediccare, Tamil Nadu, India). The DNA isolated from the blood sample of a microscopically confirmed *Theileria annulata* infected cow was used as positive control. Similarly, the DNA isolated from apparently healthy cattle with no signs of theileriosis and microscopically confirmed as negative for presence of any piroplasm during routine examination was used as a negative control.

### 2.3. In Silico Prediction of the Peptide Candidate

The amino acid sequence of *Theileria annulata* Surface Protein (TaSP) was retrieved from uniport server. The amino acids (a. a.) sequence with ID Q8WPH8_THEAN of length 314 was retrieved and used for subsequent analysis. Linear B-cell epitopes were predicted using four different prediction tools (1. ABCpred: onlinehttp://crdd.osdd.net/raghava/abcpred/ (accessed on 22 February 2022) [17]; 2. BCpred: http://crdd.osdd.net/raghava/bcepred/ (accessed on 22 February 2022) [18]; 3. IEDB: https://www.iedb.org/ (accessed on 22 February 2022) [19,20]; 4. IgPred: http://crdd.osdd.net/raghava/igpred/) (accessed on 22 February 2022). Default parameters were chosen to conduct the search, except for the ABCpred server where 16 mers were selected. Bepipred linear epitope prediction 2.0 and Kolaskar–Tongaonkar antigenicity prediction were opted for in the IEDB analysis platform for prediction of epitopes. The retrieved sequences were aligned, and a consensus sequence was derived manually.

### 2.4. In Silico Characterization of Predicted Peptide and Delineation of 3D Structure

Peptide property calculator (Ver 3.1) (https://www.biosyn.com/peptidepropertycalculator/peptidepropertycalculator.aspx) (accessed on 22 February 2022) and protoParam (https://web.expasy.org/protparam) (accessed on 22 February 2022) tools were used to characterize the peptide. The 3D structure was predicted using Phyre^2^ (http://www.sbg.bio.ic.ac.uk/~phyre2) (accessed on 22 February 2022) [21] protein modeling tool. The normal mode of modeling was chosen and the predicted structure was visualized with RasMol software (Version 2.7.5). The predicted structure was further assessed for determination of torsional angle in secondary structure, for which a .pdb file was used and Ramachandran’s plot was predicted by Ramachandran plot server (https://www.umassmed.edu/zlab/software/) (accessed on 22 February 2022).

### 2.5. Custom Synthesis of the Candidate Peptide

The peptide was custom synthesized at 95% purity by Biotechdesk Pvt. Ltd., Telangana, India. It was conjugated to BSA at N-terminus by a Cys- residue. For peptide synthesis, Fmoc chemistry was followed. Briefly, Fmoc-Ala-Wang resin was used as matrix and support. The amino acids were coupled one after another with Fmoc-amino acid residue from C-ter to N-ter. After completion, the crude peptides were cleaved from the resin by 95% trifluoroacetic acid (TFA) followed by filter separation and precipitation from diethyl ether. Finally, it was lyophilized to yield white solid powders. Peptides were analyzed and purified by reversed-phase high-performance liquid chromatography (RP-HPLC) with a linear gradient of water (0.1% TFA)–100% acetonitrile (0.1% TFA). The flow rate was maintained at 1 mL/min and injection volume was 10 µL. The separation was recorded at 220 nm. C18 column was used with dimensions 4.6 × 250 mm. Furthermore, the *m*/*z* ratio of the peptide was determined by mass spectrometry (LC–MS). LC–MS analysis was performed using an Agilent-6125B mass analyzer, keeping the flow rate at 0.2 mL/min and buffer concentration as 50% H_2_O/50% acetonitrile.

### 2.6. Western Blot and Dot Blot Analysis of the Peptide

One microgram of each BSA-conjugated peptide or BSA (fraction V) was loaded to a 12% SDS-PAGE. Here, BSA (fraction V) served as the conjugate control. The protein samples were run in quadruplicates (two for staining with CBB-R-250 and two for Western blot). The proteins were transferred to a nitrocellulose membrane (Axiva) in transfer buffer, as described before [22]. A constant current of 300 mA was applied for 1 h to transfer the proteins. The blots were incubated with 3% skimmed milk powder in PBS for overnight. The membranes were washed with PBS for 3 times and incubated with bovine plasma (positive for *T. annulata* by microscopy and PCR) at a dilution of 1:100 in PBS-T (0.05%). This was followed by washing three times with PBS-T (0.05%). Anti-bovine IgG-HRP (A5295, Sigma)/IgM-HRP (NB756, NOVUSBIO) conjugates were used at 1:2000 dilution in PBS-T (0.05%). Both blots were developed by adding 3,3′-diaminobenzidine as chromogenic substrate. Parallelly, the CBB stained gels were destained with acid methanol solution. In the Dot blot, 2.5 µg of the peptide conjugate, BSA and *E. coli* lysate, and 2.5 µL of PBS, were immobilized on a nitrocellulose membrane as “spots”. The membranes were prepared in duplicates (1 each for detection of “IgG”/“IgM”). The membrane was dried in an incubator for 1 h at 37 °C followed by blocking in 3% skimmed milk powder (in PBS) overnight. Further, the membranes were processed similarly to those of Western blot.

### 2.7. Cloning, Expression, Purification and Western Blot Analysis of rTaSP(tr)

A codon-optimized synthetic gene, which encodes the *Theileria annulata* surface protein without the signal sequence (TaSP, 20–314 aa), was available in the laboratory. The synthetic TaSP gene of 888 bp was amplified using the following set of primers: forward primer (5′-3′)—ATGAATTCGGACCATTTTTGCCCTTAG; reverse primer (5′-3′)—ATAAGCTTTTAGCAGCAATCTTCGTTAAT. The synthetic gene contains a site for BamHI (at 73–78 bp) and the reverse primer has a site for HindIII. The sequence expressing a truncated version of TaSP (44–314 aa) was derived by digesting the TaSP amplicon with BamHI and Hind III for cloning in pProExhtA expression vector. The recombinant plasmid was transformed into BL21 (DE3) cells and the protein was purified from the induced culture (with 1 mm IPTG), as described by Mishra et al. [22]. The protein was dialyzed against PBS and stored in aliquots at −20 °C until further use. For Western blot analysis, samples from the cattle with clinical signs of theileriosis and a positive diagnosis for infection by microscopy and PCR were taken as positive controls. Plasma of five healthy zebu calves (1–3 months) without any signs and history of theileriosis, and found negative for *T. annulata* by microscopy as well as Tams1 PCR, were used as negative controls. The animals are maintained at the instructional livestock farm complex of the faculty of veterinary and animal sciences, BHU, and the samples were submitted for routine surveillance. The Western blot was performed as described by Mishra et al. [22] with minor modifications. Briefly, 0.5 µg (determined by Bradford assay kit, HiMedia) of rTaSP(tr) was loaded to SDS-PAGE followed by transfer to a nitrocellulose membrane at 200 mA for 1 h. The empty spaces were blocked by buffered solution of skimmed milk powder. The membrane was washed with phosphate-buffered saline and incubated with positive or negative serums at 1:100 dilutions. Anti-bovine IgM-HRP conjugate was used for detection at 1:2000 dilution. The blot was developed with Diamino-benzidine solution.

### 2.8. Indirect Enzyme-Linked Immunosorbent Assay (ELISA)

High-binding, flat-bottom, 96-well plates (EP3, HiMedia) were used for ELISA. A total of 1 µg of either BSA-conjugated peptide (TaSP-34-BSA) or BSA (fraction V) or rTaSP(tr) in carbonate–bicarbonate buffer (pH 9.4) was coated to wells in quadruplicates for all the ELISAs. Initially, a titration assay was performed with various dilutions of known plasma to optimize the ELISA protocol for both TaSP-34-BSA and rtTaSP(tr). Plasma isolated from PCR and microscopically confirmed positive and negative blood samples were diluted in PBS at 1:100, 1:200, 1:500 and 1:1000 dilutions and used for the titration. Following coating, the plate was incubated at 4 °C for 16 h. The wells were emptied and washing was performed 4 times with 100 µL of PBS. For blocking, 1% porcine gelatine in PBS was used. Various dilutions of both positive and negative plasma samples were probed to blocked wells post washing with PBS. Bovine anti-IgM-HRP conjugate was used at 1:5000 dilutions in PBS-T (0.05%) for tagging any bound bovine IgM to the BSA-conjugated TaSP-34 peptide. After 4 washes with PBS-T (0.05%), 100 µL of TMB substrate solution (ML168, HiMedia) was added to the wells. The enzymatic reaction was stopped by adding 1N of H_2_SO_4_. Absorbance at 450 nm (OD_450_) was recorded by a microplate reader (ADX-Alta 110 microplate reader, Athenese Dx, Tamil Nadu, India). The values (OD_450_) obtained in the BSA wells were subtracted from their peptide counterparts for different dilutions of both positive and negative samples. Similarly, the serum control wells were used for normalization in rTaSP(tr) ELISA. The responses obtained were compared and statistically analyzed. The dilution at which maximum significant difference was marked between positive and negative samples was considered as the screening dilution for test plasma samples. Mean ± 2 standard deviations of the OD_450_ of five negative samples were calculated for determining the cut-off value. The test samples were analyzed similarly to the method described above. A similar procedure was adopted for screening with rTaSP(tr) antigen.

### 2.9. Comparison of ELISA with PCR and Statistical Analysis

The efficiency of the TaSP-34-BSA was compared with rTaSP(tr) as an ELISA candidate. The TaSP-34-BSA was assessed for various parameters like specificity, sensitivity, positive predictive value (PPV), negative predictive value (NPV), etc. with reference to rTaSP(tr). The PPV is the ratio of the patients truly diagnosed as positive to all those who had positive test results. It predicts the proportion of the test population who gave positive test result and are actually infected or diseased. The NPV is the ratio of animals truly diagnosed as negative to all those who had negative test results. It predicts the likelihood of an animal to be truly healthy, in case of a negative test result. An online-based server, MedCalc^®^ (https://www.medcalc.org/calc/, accessed on 22 February 2022), was used for calculation of various performance parameters like specificity, sensitivity and accuracy. The agreement between the tests was determined by calculation of Cohen’s kappa coefficient (“κ”). “IDoStatistics” server (https://idostatistics.com/, accessed on 22 February 2022) was used for the calculation of “κ”. All the statistical evaluations were performed using Graphpad prism, Version 8.0. Unpaired *t*-test was performed for comparison between positive and negative plasma samples.

## 3. Results

### 3.1. Prediction and Synthesis of Candidate Antigen

**Figure 1:**
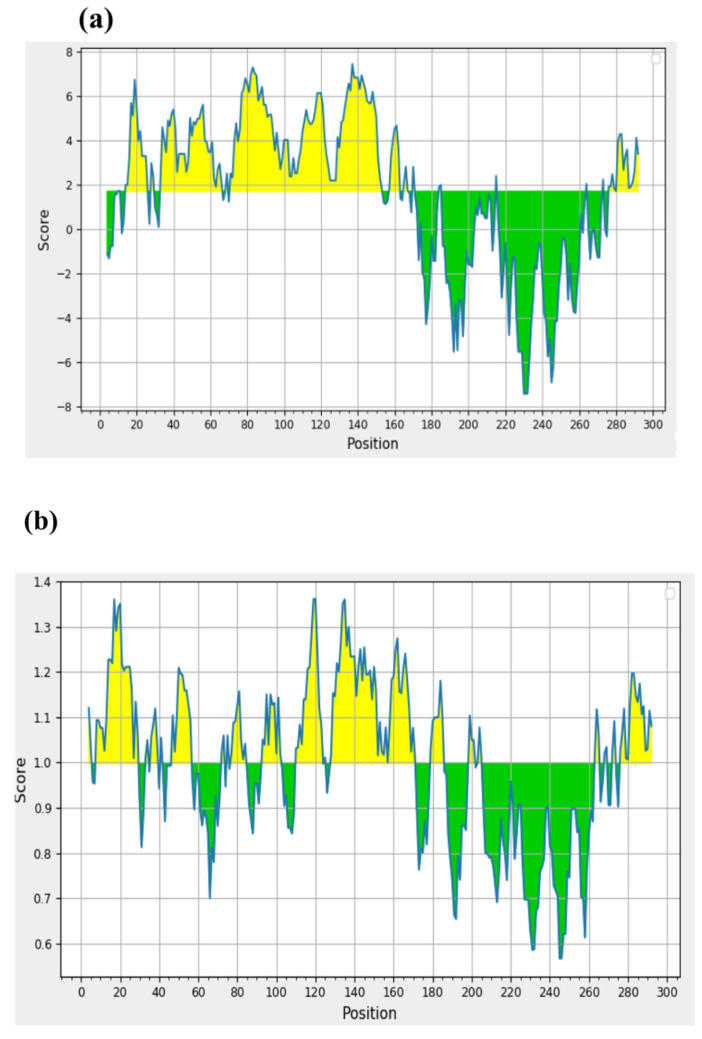

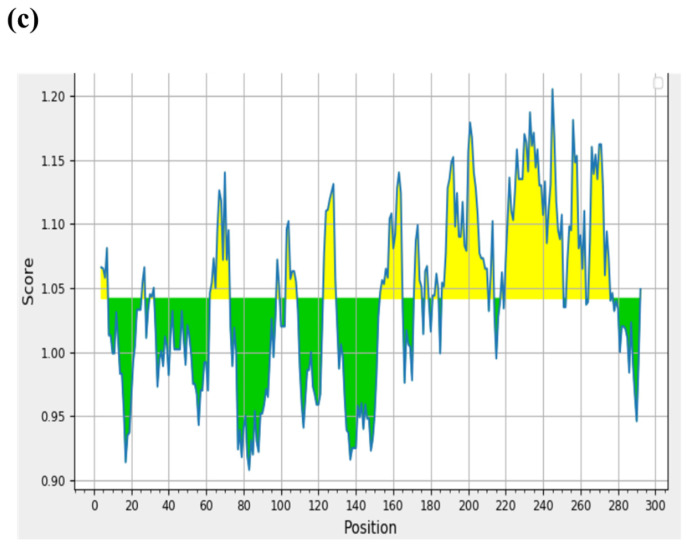
Different plots of TaSP-34 related to antigenicity, as predicted by IEDB server. (**a**) Parker hydrophilicity; (**b**) Chou–Fasman Beta-Turn Prediction; (**c**) Kolaskar–Tongaonkar antigenicity plot.

**Figure 2:**
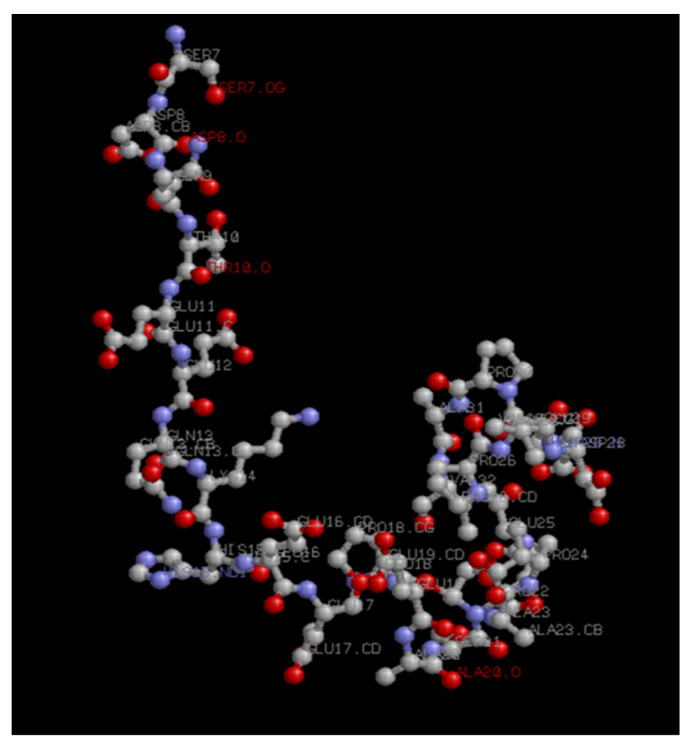
Predicted three-dimensional structure of TaSP−34. The .pdb file was visualized in RasMol viewer software.

**Figure 3:**
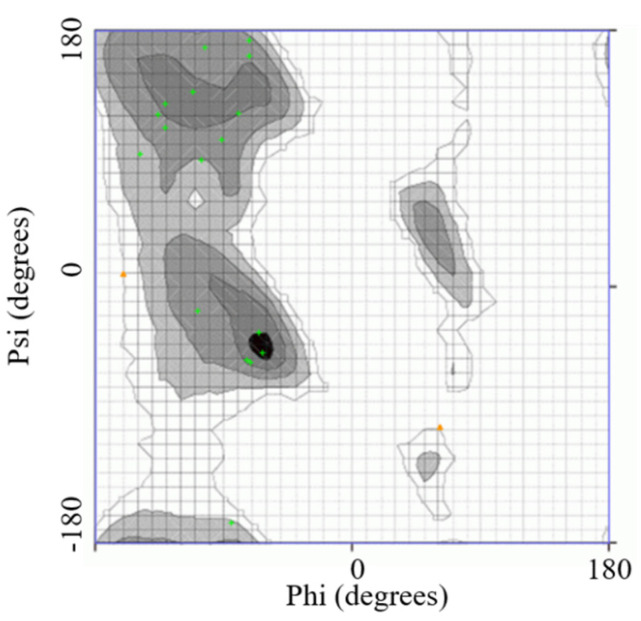
The presented Ramachandran plot accurately predicted coordinates of 3D structure as most of the residues fall in the sterically favorable zone. Green dots indicate residues in the appropriate region.

**Figure 4:**
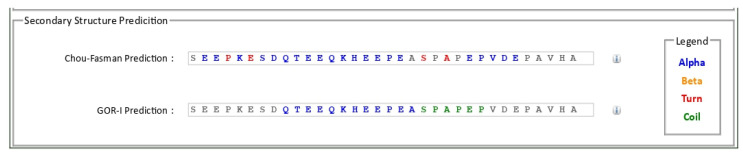
The propensity of different residues in TaSP-34 to adopt various secondary structures are being represented.

**Figure 5:**
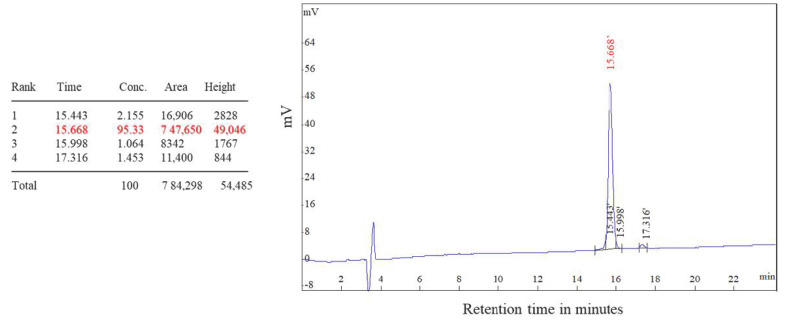
HPLC chromatogram of the peptide with properties of different peaks observed. In total, 95.33% of the preparation corresponds to the desired peptide.

**Figure 6:**
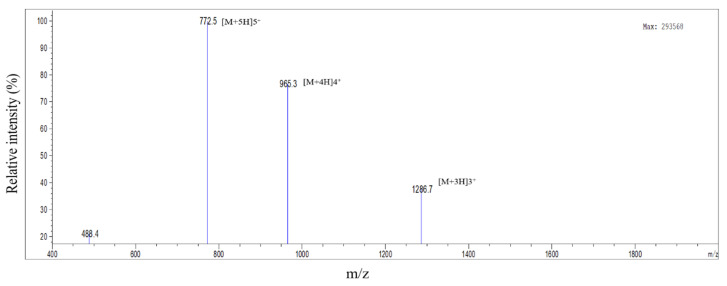
LC–MS analysis of the peptide. The *m*/*z* ratio was plotted against relative intensity.

Different epitopic regions were predicted by the four prediction servers. ABCpred predicted 20 potential antigenic stretches above the threshold value (0.51). Bepipred 2.0 resulted four antigens, and Kolaskar–Tongaonkar antigenicity predicted a total of eight sequences. Five antigens were identified by IgPred. Consensus was inferred by aligning the predicted sequence (Table 1). It was observed that the region from 70 to 110 encompasses most of the predicted sequences. A stretch of 34 a.a. from 74 to 108 was finalized as the candidate peptide (TaSP-34). The peptide was well demarcated within the hydrophilicity plot (Figure 1a). The residues in the predicted peptide with the ability to form β-turns are presented in Figure 1b. The Kolaskar–Tongaonkar plot describes the stretch as potentially immunogenic (Figure 1c). Chemically, the peptide is an acidic species. TaSP-34 has a theoretical isoelectric pH (pI) of 4. The neutral and acidic amino acid contents are maximum and equal in the peptide (Table 2). The predicted 3D structure of the peptide appears to be an “inverted comma”-like projection (Figure 2). Most of the residues were exposed to the outside and accessible to an exterior solvent, which is highly desirable for such detection candidates. Ramchandran’s plot suggests that the predicted structure is accurate as about 90% of the residues fall in the favorable zone of the plot (Figure 3). Moreover, the tendencies of different constituent amino acids to adopt a suitable 2° structure are represented in Figure 4. Most of them adopt an α-helix under the proper folding condition. The synthesized peptide was purified by RP-HPLC. The chromatogram can be referred to for the abundancy (95%) of the desired peptide in the preparation (Figure 5). Further, the peptide was confirmed by estimation of the *m*/*z* ratio in LC–MS analysis (Figure 6).

### 3.2. Screening of Blood Samples by PCR and Microscopy

**Figure 7:**
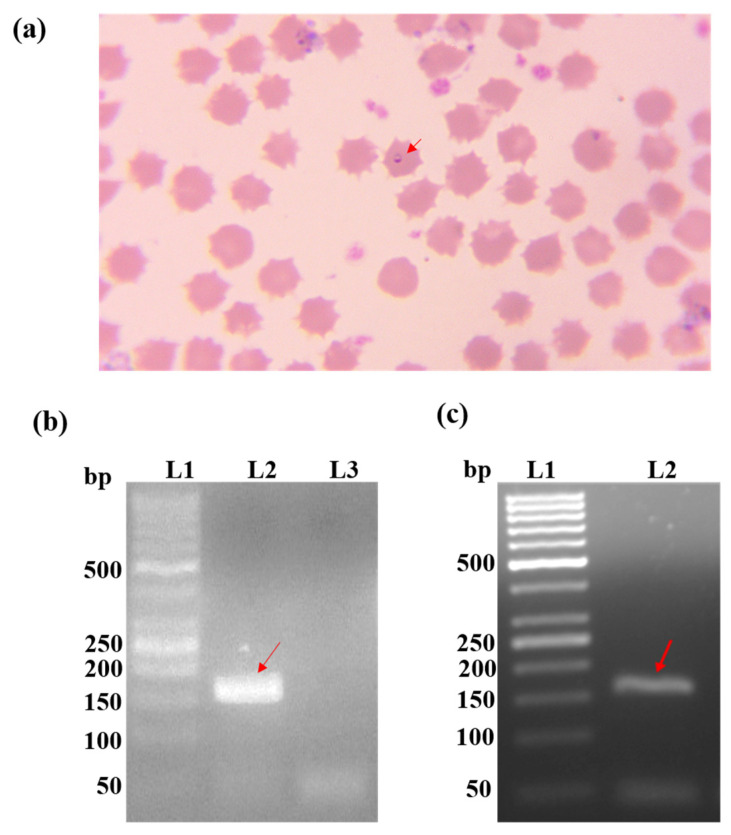
(**a**) Giemsa-stained blood smear showing presence of piroplasm (red arrow) of *Theileria annulata* in bovine erythrocytes. In microscopically positive samples, 0.01–0.03% of RBCs were found to harbor piroplasm. (**b**) Tams 1 PCR of positive and negative controls: lane 1 contains 50 bp ladder; lane 2 and lane 3 correspond to the positive and negative controls, respectively. The arrow mark indicates the presence of the amplicon of desired length (156 bp) in the positive control lane. (**c**) Agarose electrophoresis of Tams1 PCR screening of a test sample: lane 1 contains 50 bp ladder and lane 2 contains amplicon of the test sample. Red arrow indicates the 156 bp amplicon. Similarly, the samples found positive by Tams 1 PCR showed an amplicon of the same size (not shown in the figure).

The blood samples were screened for *T. annulata* infection by microscopy and PCR. A total of 18 samples were found to have many piroplasms in the erythrocytes (Figure 7a). Presence of a PCR amplicon of 156 bp (Figure 7b) is an indication of Tams1 and, thus, the *T. annulata* genome. Forty-six (46) samples were found to be positive by Tams1 PCR (Figure 7c). All eighteen samples found positive by microscopy were also positive in Tams1 PCR. A total of 14% of the samples were found to be positive by microscopy, while PCR detected 35.9% of the samples as positive for *T. annulata* infection. In our case, microscopy was found to be 39% sensitive and 100% specific to Tams1 PCR.

### 3.3. Western Blot and Dot Blot Analysis

**Figure 8:**
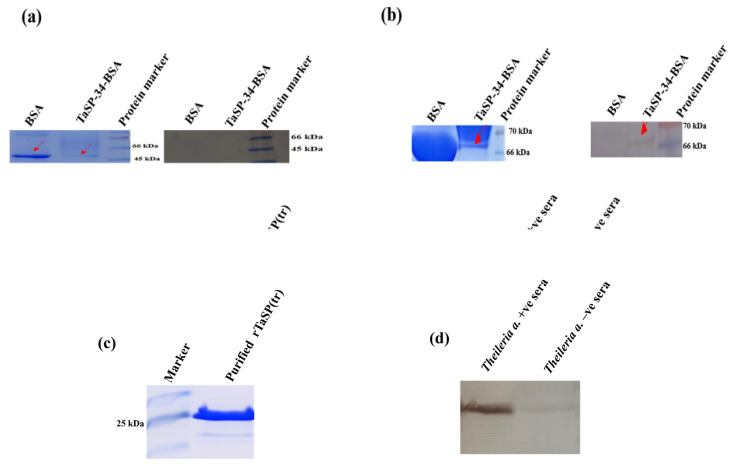
Western blot analysis: (**a**) Membrane with the immobilized protein/peptide incubated with *T. annulata* positive plasma and anti-bovine IgG-HRP was used for detection of any interaction. (**b**) Anti-bovine IgM-HRP was used as detection antibody. In both cases, the destained SDS-PAGE image is shown to the left of the corresponding blots. (**c**) Western blot analysis of rTaSP(tr); affinity-purified rTaSP(tr) was analyzed on a 12% SDS-PAGE. The size of the truncated protein corresponds to ~27 kDa. (**d**) The membrane-immobilized rTaSP(tr) captured bovine IgM against *T. annulata* at a dilution of 1:100 of the positive plasma sample. One of the samples used as a negative control in the ELISA was used at 1:100 dilution. Anti-bovine IgM-HRP conjugate at 1:500 dilutions served as detection antibody for the Western blot.

**Figure 9:**
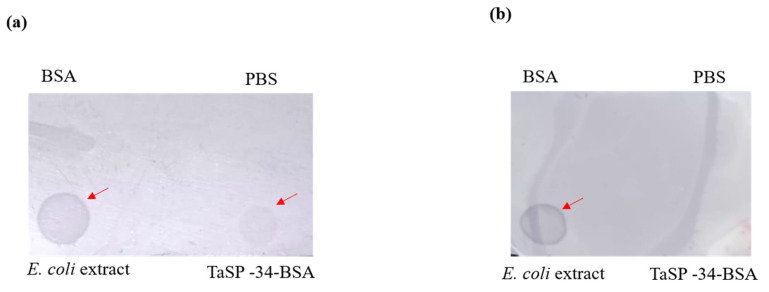
Dot blot analysis: (**a**) TaSP conjugate and *E. coli* extract gave positive spots when detected by anti-bovine IgM-HRP conjugate. (**b**) The TaSP-34-BSA did not react to bovine IgG. In both cases, PBS and BSA did not react to bovine IgM or IgG, or to the detection conjugates.

Neither BSA nor the peptide conjugate (TaSP-34-BSA) captured bovine IgG (Figure 8a), but TaSP-34-BSA was found to interact with bovine IgM. The positive band was indicated by a red arrow mark (Figure 8b). SDS-PAGE showed the presence of both proteins (BSA and TaSP-34-BSA). Similarly, the interaction of rTaSP(tr) with bovine IgM against *T. annulata* was quite evident, and the Western blot is shown in Figure 8c,d. The Dot blot showed a similar result to that of the Western blot in which the peptide conjugate did bind to IgM (Figure 9a), but IgG (Figure 9b) of *T. annulata* positive plasma was not retained on the membrane. Presence of bovine IgG/IgM in the plasma was indicated by the spot developed on the *E. coli* extract impregnated site on both the membranes.

### 3.4. Performance of the TaSP-34-BSA in Indirect ELISA

**Figure 10:**
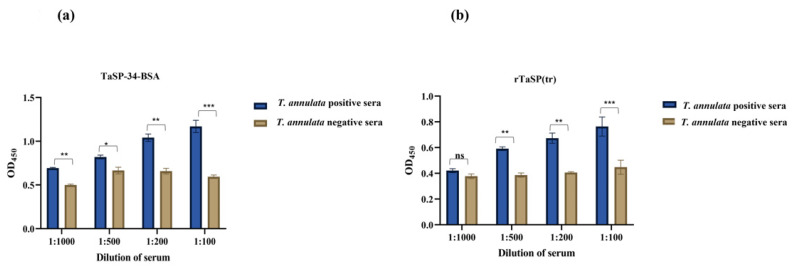
Optimization of plasma dilution for IgM-ELISA of TaSP-34-BSA and rTaSP(tr): (**a**) The IgM-ELISA of different dilutions of plasma from positive and negative samples using TaSP-34-BSA as the detection antigen. (**b**) The ELISA data of rTaSP(tr) against IgM at different dilutions of sera of positive and negative samples. The values are Mean ± SD of four replicates. A *p* value ≤ 0.05 was considered significant, and annotations are * for *p* < 0.05, ** for *p* < 0.01, *** for *p* < 0.001 and ns—not significant.

**Figure 11:**
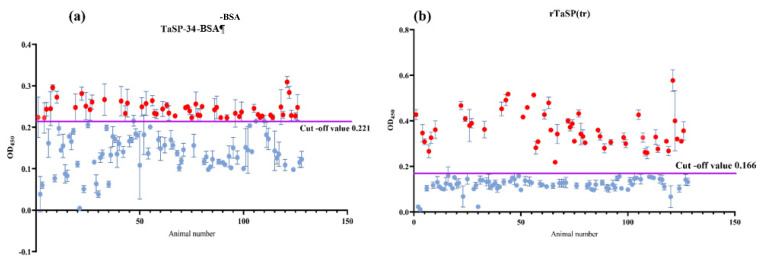
Screening of the 128 test samples by TaSP-34-BSA based indirect plate ELISA: (**a**) The TaSP-34-BSA peptide detected a total of 52 plasma samples positive for IgM against *T. annulata* (Red dots). (**b**) A total of 51 plasma samples were positive (Red dots) for IgM against *T. annulata* by rTaSP(tr) ELISA.

The optimization of the dilution of plasma suggested that TaSP-34-BSA and rTaSP(tr) can differentiate between positive and negative samples at all four dilutions. The differences in OD_450_ between positive and negative samples were statistically significant in all test dilutions for both antigens (Figure 10a,b). The dilution 1:100 was used invariably for testing of the rest of the samples. All the 128 samples were screened for retention of IgM with anti-bovine IgM-HRP. The cut-off OD_450_ values for TaSP-34-BSA and rTaSP(tr) are 0.221 and 0.166, respectively. The OD_450_ was plotted against animal number and represented graphically in Figure 11a for TaSP-34-BSA and Figure 11b for rTaSP(tr). The known sera of three cattle with *Babesia bigemina* (confirmed by microscopy) infection were also analyzed to check for any cross-reactivity. All were below the cut-off values.

When compared with rTaSP(tr)-based ELISA as a reference test, the TaSP-34-BSA is 98.04% sensitive and 97.44% specific (Table 3). Furthermore, the peptide is 100% sensitive and 92.77% specific with regards to Tams1 PCR (Table 4). Again, the peptide is 100% specific and 69% specific in comparison with conventional microscopy (Table 5). The positive predictive value of the peptide-based ELISA is 96.15% and the negative predictive value is 98.70%. The accuracy of the test is 97.66%. All these parameters were calculated by considering the rTaSP(tr)-based ELISA as the reference test. Cohen’s kappa value was found to be 0.95 and the agreement was 97.65%, suggesting that the agreement in this test is almost a perfect agreement. The positive likelihood ratio (PLR) and negative likelihood ratio (NLR) of the test is 37.75 and 0.02, respectively.

As per our observation with 128 samples, the rTaSP(tr) detected 39.84% as positive but the TaSP-34-BSA detected 40.62% as positive for IgM against *T. annulata* (Table 6).

## 4. Discussion

Tropical theileriosis has been a major cause of economic loss in the dairy industry [23]. The diagnosis of the disease is mostly limited to microscopic identification of developmental intermediates of *T. annulata.* Most often, the examiner skips the carrier stages and low load of pathogens while screening the Giemsa-stained blood smears [24,25]. Apart from the treatment of the infected animals, a holistic approach for the control and eradication of theileriosis shall be adopting effective vaccination, controlling the tick population and implementing other preventive measures [2]. However, an effective control strategy needs a detailed prevalence study of the disease and demarcation of the endemic regions. Serology has been widely used for prevalent studies. Several test platforms are available but with few detection candidates for the diagnosis of IgG against *T. annulata* with variable specificity and sensitivity [10,11,12,26]. In the present study, we tried to explore the diagnostic potential of a synthetic peptide antigen for the screening of antibodies against *Theileria*. TaSP has been established as the most efficient serological candidate for the detection of tropical theileriosis [14,27]. In the current study, TaSP was used as a template to predict an immunodominant linear epitope, which may bind to the cognate paratopes of bovine antibodies. Several prediction modules were used for the prediction. These servers use different algorithms to compute epitopic candidates of different lengths. So, a consensus of amino acids was necessary to predict an immunodominant epitope. A stretch of the TaSP, spanning most of the common amino acids predicted as an epitope/part of the epitope by different servers, was mapped between N-terminus-70-110. The sequences N-74 to 108 were selected as the peptide candidate (TaSP-34). The size of the peptide was kept below 50 amino acids as a larger peptide is difficult to synthesize [28]. The peptide was custom synthesized. The inability of peptides to give an optimum signal in serology has been a limiting factor for peptide-based diagnostic. It mostly happens due to the small size, which compromises its presentation to antibodies [29]. BSA was conjugated to the peptide to address this issue. As a homologous protein, BSA was expected to give the least background signal while testing bovine plasma. LC–MS is widely used with HPLC to confirm the peptide sequence and its purity. The peptide was purified at 95% purity to avoid any cross-reactivity in the assays. The sequence of the peptide was confirmed by LC–MS analysis. The synthesized peptide was analyzed for immunoreactivity with *T. annulata* positive sera by Western blot and Dot blot. Both anti-bovine IgM- and IgG-HRP conjugates were used to check the efficiency of the peptide in binding to these two classes of antibodies. This could have paved the way to devise two different platforms for screening of early infection (IgM) and any previous exposure to the pathogen (IgG). In Western blot, the peptide only interacted with bovine IgM. Surprisingly, the TaSP-34-BSA migrated differentially in SDS-PAGE. The gel used for the IgM blot showed a band at ~66 kDa (TaSP-34-BSA), but the one transferred for IgG migrated until 50 kDa. It was suspected that this anomalous behavior of the peptide might be due to cleavage of the BSA, which might have affected the immunoreactivity, as observed with IgG. But the results were repeated in Dot blot, which confirmed the observed electrophoretic pattern of the peptide to be irrelevant with respect to the differential binding to IgG and IgM in the Western blot analysis. TaSP has been established as an effective indirect ELISA candidate for the detection of theileriosis [14,27]. Epitope mapping of TaSP suggested the initial 50 amino acids (from N-terminus) to be non-immunogenic. So, a truncated TaSP (44-314) was used as a reference candidate for the evaluation of TaSP-34-BSA based ELISA. Statistically, the plasma diluted at different dilutions in PBS gave a significant difference in OD_450_ between a known *T. annulata* positive sample and negative sample upon detection with TaSP-34-BSA and rTaSP(tr). Further, it was ascertained that there was no cross-reactivity between *Babesia b.* interacting antibodies and TaSP-34-BSA. TaSP-34-BSA ELISA is equally sensitive to Tams1 PCR, but its specificity is 92%. The lower specificity might be explained as observed before while comparing two heterogenous tools [6,30,31]. Again, PCR targets amplification of the nucleic acids, thus indicating active infective stages, while ELISA/serological methods target antibodies that need a certain period (7 days for both IgM and IgG in a parasitic infection [32]) until the circulating antibodies appear from the date of initial exposure to the pathogen. So, this could also be a reason for the differential observations in performance of both of the platforms. However, the TaSP-34-BSA based ELISA is equally competent to that of the recombinant protein, i.e., rTaSP(tr)-based ELISA. The kappa value between the two tests suggests a near-perfect agreement. Thus, it can be a promising candidate for future generation diagnostics. Again, such a peptide-based approach may replace recombinant proteins, the use of which is often limited by poor shelf life, cross-reactivity and a relatively higher production cost. Several approaches have been investigated for the diagnosis of *T. annulata* infection in cattle. The most practiced routine microscopy is limited by sensitivity and, most often, an inability to detect carrier stages. Specialized techniques like xenodiagnosis are not suitable for high-throughput screening [33]. Serological techniques like the complement fixation test and IFAT are not cost-effective. Moreover, cross-detection between different species of Theileria limits the potential of such platforms. The IFAT was found to be 70.7% sensitive and 81.85% specific with respect to TaSP ELISA [6]. Another serological test, Dot-ELISA, was found to be 95.8% sensitive and 80% specific in comparison with TaSP ELISA [27]. Molecular assays like PCR and real-time quantitative assays are effective in terms of sensitivity and specificity, but the involvement of sophisticated equipment and ineptitude to detect protective antibodies/serological responses have been the drawbacks of such tools. So, the performance of TaSP-34-BSA with 98% specificity and 97% sensitivity in comparison with TaSP ELISA establishes it as a potential diagnostic candidate for tropical theileriosis.

## 5. Conclusions

The results of the study indicate that peptides can replace the recombinant proteins for the diagnosis of IgM against *T. annulata*. Developing serological tests like ELISA, lateral flow assay and dipstick assays based on such predicted peptides may result in new-generation pen-side tests. Again, similar approaches can be made to derive new antigenic peptides from different immunogenic transmembrane proteins, and the other domain (C-terminus) of the TaSP can also be considered for additional candidates. Also, the efficiency of the unconjugated TaSP-34 peptide (without BSA) may be further evaluated as an ELISA candidate.

## Figures and Tables

**Table 1:** Predicted antigenic sequences (common sequences are highlighted in yellow).

Server Used for Prediction	Position in TaSP	Predicted Immunogenic Sequence
**ABCpred**	**89**	HEEPEASPAPEPVDEP
**ABCpred**	**83**	QTEEQKHEEPEASPAP
**BCpred**	**75–93**	EEPKESDQTEEQKHEEPE
**BEPIPRED**	**85**	EEQKHEEPEASPAPEPVDEP
**BEPIPRED 2.0**	**75**	EEPKESDQTEEQKHEEPEA
**IEDB**	**80–86**	ESDQTEE
**IEDB** **(Kolaskar–Tongaonakar)**	**103–108**	EPAVHA
**IgPred**		QPSTEPEELQPETVTVEVPEPVTSEEPKESDQTEEQKHEEPEASPAPEPVDEPAVHATESTPTKASSSGD
**Candidate**	**74–108**	SEEPKESDQTEEQKHEEPEASPAPEPVDEPAVHA

**Table 2:** Chemical properties of TaSP-34.

Parameters	Value	Prediction Tool
Number of residues	34	Peptide Property Calculator—Version 3.1
Molecular weight	3754 g/mol	-do-
Acidic a.a.(%)	35.29	-do-
Basic a.a.(%)	11.76	-do-
Neutral a.a. (%)	35.29	-do-
Hydrophobic a.a.(%)	17.65	-do-
pI (theoretical)	4.11	protoParam
Chemical formula	C_157_H_238_N_42_O_65_	-do-
Instability index	125.17	-do-
Aliphatic index	28.82	-do-
Grand average of hydropathicity (GRAVY)	1.774	-do-

**Table 3:** Diagnostic performance of TaSP-34-BSA indirect ELISA with reference to rTaSP(tr) indirect ELISA for the detection of anti-*T. annulata* IgM of bovines.

**As Detected by rTaSP(tr) ELISA**							**Low 95% C.I.**	**High** **95%** **C.I.**
**As Detected by TaSP-34-BSA ELISA**	**Positive**	50	02	**Total 128**				
	**Negative**	01	75		**Sensitivity**	98.04%	89.34%	99.95%
					**Specificity**	97.44%	91.04%	99.69%
					**Accuracy**	97.66%	93.00%	99.51%
					**† PPV**	96.15%	86.18%	98.97%
					**‡ NPV**	98.70%	91.61%	99.81%

Kappa value: 0.95, † PPV—Positive Predictive Value, ‡ NPV—Negative Predictive Value.

**Table 4:** Relative sensitivity, specificity and accuracy value of TaSP-34-BSA indirect ELISA for the detection of *T. annulata* infection compared with Tams1 PCR.

**As Detected by Tams1 PCR**							**Low 95% C.I.**	**High** **95%** **C.I.**
**As Detected by TaSP-34-BSA ELISA**	**Positive**	46	06	**Total 128**				
	**Negative**	0	76		**Sensitivity**	100%	92.13%	100%
					**Specificity**	92.77%	84.93%	97.3%
					**Accuracy**	95.31%	90.08%	98.26%
					**† PPV**	88.24%	77.63%	94.19%
					**‡ NPV**	100%	95.32%	100%

Kappa value: 0.90, † PPV—Positive Predictive Value, ‡ NPV—Negative Predictive Value.

**Table 5:** Relative sensitivity, specificity and accuracy value of TaSP-34-BSA indirect ELISA for the detection of *T. annulata* infection as compared with microscopy.

**As Detected by Microscopy**							**Low 95% C.I.**	**High** **95%** **C.I.**
**As Detected by TaSP-34-BSA ELISA**	**Positive**	18	34	**Total 128**				
	**Negative**	00	76		**Sensitivity**	100%	80.49%	100%
					**Specificity**	69.37%	59.91%	77.77%
					**Accuracy**	73.44%	64.91%	80.85%
					**† PPV**	33.33%	27.43%	39.81%
					**‡ NPV**	100%	95.32%	100%

Kappa value: 0.38, † PPV—Positive Predictive Value, ‡ NPV—Negative Predictive Value.

**Table 6:** Percentage of the samples detected positive by different tests.

Total No. of Samples Examined	Positive for *Theileria annulata* Infection as Detected by Different Tests				
	Microscopy	Tams1 PCR	rTaSP(tr)-Based ELISA	TaSP-34-BSA ELISA	Both TaSP-34-BSA ELISA and rTaSP(tr)-Based ELISA
128	18 (14.06%)	46 (35.93%)	51 (39.84%)	52 (40.62%)	50 (39.06%)

## Data Availability

Data are contained within the article.
